# Hundreds of novel composite genes and chimeric genes with bacterial origins contributed to haloarchaeal evolution

**DOI:** 10.1186/s13059-018-1454-9

**Published:** 2018-06-07

**Authors:** Raphaël Méheust, Andrew K. Watson, François-Joseph Lapointe, R. Thane Papke, Philippe Lopez, Eric Bapteste

**Affiliations:** 1 Sorbonne Universités, UPMC Univ Paris 06, Institut de Biologie Paris Seine, Centre National de la Recherche Scientifique, Unité Mixte de Recherche 7138 Evolution Paris Seine, 75005 Paris, France; 2 Department of Molecular and Cell Biology, University of Connecticut, Storrs, CT 06269 USA; 3 Département de sciences biologiques, Université de Montréal, Montréal, Québec Canada

## Abstract

**Background:**

Haloarchaea, a major group of archaea, are able to metabolize sugars and to live in oxygenated salty environments. Their physiology and lifestyle strongly contrast with that of their archaeal ancestors. Amino acid optimizations, which lowered the isoelectric point of haloarchaeal proteins, and abundant lateral gene transfers from bacteria have been invoked to explain this deep evolutionary transition. We use network analyses to show that the evolution of novel genes exclusive to Haloarchaea also contributed to the evolution of this group.

**Results:**

We report the creation of 320 novel composite genes, both early in the evolution of Haloarchaea during haloarchaeal genesis and later in diverged haloarchaeal groups. One hundred and twenty-six of these novel composite genes derived from genetic material from bacterial genomes. These latter genes, largely involved in metabolic functions but also in oxygenic lifestyle, constitute a different gene pool from the laterally acquired bacterial genes formerly identified. These novel composite genes were likely advantageous for their hosts, since they show significant residence times in haloarchaeal genomes—consistent with a long phylogenetic history involving vertical descent and lateral gene transfer—and encode proteins with optimized isoelectric points.

**Conclusions:**

Overall, our work encourages a systematic search for composite genes across all archaeal major groups, in order to better understand the origins of novel prokaryotic genes, and in order to test to what extent archaea might have adjusted their lifestyles by incorporating and recycling laterally acquired bacterial genetic fragments into new archaeal genes.

**Electronic supplementary material:**

The online version of this article (10.1186/s13059-018-1454-9) contains supplementary material, which is available to authorized users.

## Background

Haloarchaea (also called Halobacteria) is an archaeal class in which all members thrive in oxygenated hypersaline environments using aerobic respiration and reduced carbon sources. This lifestyle is in distinct contrast with the physiology of their methanogenic ancestors, which were autotrophic, and lived in oxygen-free habitats [1]. Furthermore, Haloarchaea adapted to extreme osmotic challenges by adopting a salt-in strategy making their cytosolic salinity equal to that of their environment – halophilic methanogens use compatible solutes to balance their osmotic pressures [2]. These major lifestyle transitions (a process we termed “haloarchaeal genesis”) implied that Haloarchaea faced at least two major issues. It involved numerous genetic events to transform their physiology, as well as amino acid optimizations, which allowed their proteins to remain soluble, resulting in lower isoelectric points than their homologs outside this group [3]. While the latter changes can result from point mutation, abundant lateral gene transfers (LGT) from bacteria have repeatedly been invoked to explain the evolution and adaptation to oxygenic lifestyle of this archaeal lineage [4].

Phylogenetic studies, largely focused on the acquisition of full-sized genes by Haloarchaea from bacterial donors, proposed either a sudden and massive introgressive process [5, 6], or a more gradual and piecemeal process [7, 8] to explain the gains of a thousand gene families with bacterial origins in the haloarchaeal group [5, 6]. Integrative modeling of gene and genome evolution in the archaea has also suggested that, though gene families are largely vertically transmitted within archaea, LGT has had a significant impact on archaeal genome evolution, outnumbering expansion of genomes by duplication of existing archaeal gene families in the majority of branches of the archaeal tree, including in the haloarchaea where rates of LGT were particularly high [9]. Importantly, in addition to these recognized LGTs, the evolution of novel genes within the group could also explain how Haloarchaea arose and thrive, and this is supported by the discovery of gene families corresponding to potential de novo origins in the Haloarchaea [9]. This type of change has rarely been assessed because little is known about the origins of novel genes in prokaryotic genomes [10, 11]. There are a range of different mechanisms that can produce novel genes, including de novo genes, synthesized either partly or completely from non-coding DNA [12], from the divergence of an existing protein-coding sequence beyond the point at which it is recognizable as a homologue (e.g. following gene duplication events), or by fusion or fission of existing protein-coding sequences [13]. New genes have been shown to be able to fulfil crucial roles in biological processes after relatively short evolutionary times for different lineages, highlighting their potential importance in biological transitions [14]. Yet, events of gene remodeling leading to the creation of novel genes, possibly contributing to haloarchaeal genesis and to the success of their descendants, remain to be systematically investigated.

Gene remodeling has been described in prokaryotes, mainly resulting from the fusion and fission of full-sized genes [15]. These two processes produce detectable composite genes, i.e. genes composed of dissociable/associable parts, called components. Moreover, the transfer of DNA fragments, i.e. subgenic regions shorter than entire genes (e.g. domains), has also been reported for prokaryotes [16, 17]. This latter process of genetic acquisition could, in principle, be followed by genomic rearrangements, when the laterally acquired domains combine with genetic material already present in their new host genomes. In eukaryotes, this process led to the evolution of a remarkable class of composite genes, i.e. the symbiogenetic genes (S-genes). S-genes emerged when subgenic fragments from mitochondrial or chloroplastic endosymbionts merged together or merged with the eukaryotic host DNA in the nucleus [18]. In the case of photosynthetic eukaryotes, 67 such novel families of S-genes with likely adaptive functions were recently reported [18].

The detection of gene remodeling, including the fusion and the recycling of domains derived from heterologous proteins, can be studied effectively using network approaches [18, 19]. Here, we used sequence similarity networks [19] that rely on both full and partial (e.g. protein domain) pairwise similarity values to analyze similarity between sequences and to test whether gene remodeling was involved in the emergence of Haloarchaea and in their subsequent evolution. We report the creation of hundreds of novel composite genes, both early in the evolution of Haloarchaea (during haloarchaeal genesis) and later in diverged haloarchaeal groups. Based on the taxonomic assignment of the components of the composite genes, we distinguish three classes of composite genes, exclusively found in Haloarchaea. First, we identified class I composite genes, which are formed from unique associations of DNA components from archaeal genomes (i.e. ARC-ARC and ARC-HALO composite genes). Second, we identified class II composite genes, which are derived from unique combinations of prokaryotic DNA, since these components cannot be confidently assigned either to a bacterial or an archaeal host (i.e. PROK-PROK, PROK-ARC, PROK-HALO composite genes). Third, we identified chimeric composite (ChiC) genes in Haloarchaea, which are made up of (at least one) component from bacterial genomes (i.e. BAC-HALO, ARC-BAC, and PROK-BAC composite genes). Importantly, these latter genes constitute a different gene pool from the laterally acquired bacterial genes detected by Nelson-Sathi [5]. Hence, haloarchaeal ChiC genes reveal an additional substantial bacterial contribution during the evolution of Haloarchaea. Many of these novel composite genes, i.e. ChiC genes and class I and II composite genes, were likely advantageous for these hosts. They showed significant residence times in haloarchaeal genomes, as assessed by their taxonomic distributions, consistent with a long phylogenetic history involving vertical descent, and by the optimized isoelectric points of composite and ChiC genes, which allow their encoded proteins to operate in salty environments. Importantly, while the novel ChiC genes are largely involved in metabolic functions such as carbohydrate metabolism which was absent in the haloarchaeal ancestors, some composite and ChiC genes, involved in redox reactions, may have also played a role in the adaptation of Haloarchaea to an oxygenic lifestyle and salty environment.

## Results and discussion

### Detection of composite genes and ChiC genes in Haloarchaea

We clustered 1,816,486 archaeal proteins from 802 genomes into 49,269 families. In total, 6417 families (including 132,458 proteins) were found in at least three different haloarchaeal genomes and were exclusive to Haloarchaea. These 132,458 proteins were further aligned over an extended bacterial database of 7,239,663 sequences from 2078 bacterial genomes in order to remove families with full-length similarities to bacterial proteins. A total of 5558 families were retained from this additional screen and are therefore good candidates for novel, clade-specific genes because they likely originated during or after the emergence of Haloarchaea, since homologs of these Haloarchaeal genes cannot be found in any other taxa. We tested whether these exclusive haloarchaeal genes were composite, i.e. whether some of their constitutive subgenic regions, called components, also matched with distinct gene families (particularly in 7,239,663 bacterial sequences). We combined this detection of component and composite genes with an additional step of domain annotation (see “Methods”). This protocol returned 320 composite gene families, exclusive to Haloarchaea.

**Fig. 1:**
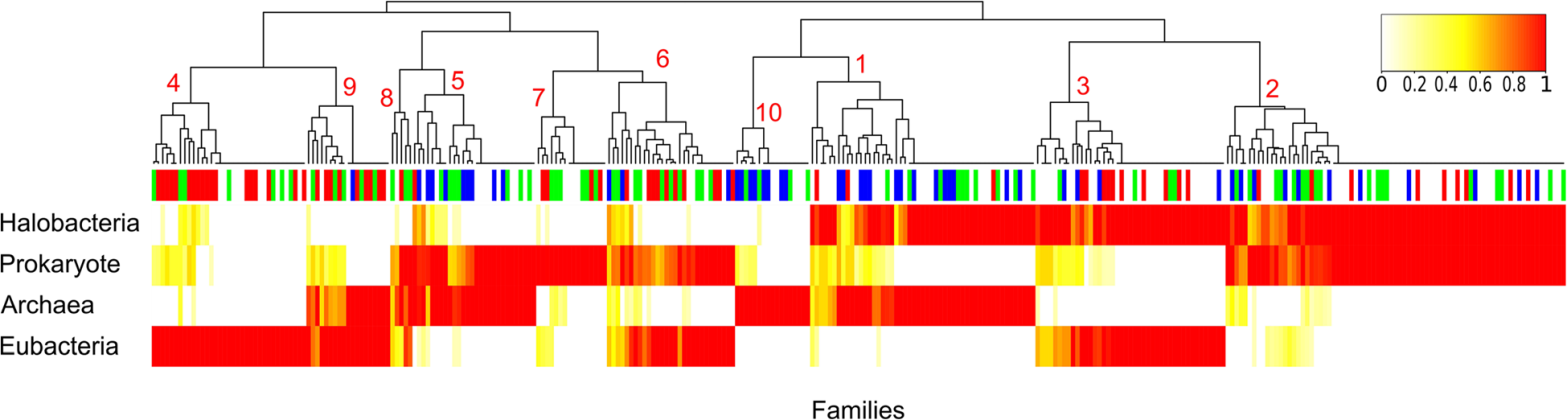
Hierarchical clustering of composite genes families according to their component origins (as assigned by BLAST). The *heatmap* represents the ratio of genes in a given family (columns) which have at least one component of a given origin (haloarchaeal, archaeal, bacterial or prokaryotic, rows). A *white tick* corresponds to the absence of components from a given origin in every gene in a given composite gene family. *Colored ticks* correspond to the presence of at least one component of a given origin at a given percentage (*red* for 100% of the genes in a composite gene family). The heatmap is hierarchically clustered by gene families. The *colored top bar* indicates the functional annotation of the composite gene families according to COG categories (*red*: metabolism, *blue*: information storage and processing, *green*: cellular processes and signaling, *white*: poorly characterized). The Euclidean distance and the complete linkage method were used for the hierarchical clustering

We classified these families into three major groups, based on the taxonomic assignation of their components (see “Methods,” Table 1 and Fig. 1). First, there were 68 families of class I composite genes that exclusively combined components of archaeal origin (clusters 1 and 10 in Table 1, derived from the heatmap in Fig. 1), yet in a combination only observed within Haloarchaea. Second, there were 126 composite gene families, which presented at least one component of bacterial origin (clusters 3, 4, 6, and 9 in Table 1, derived from the heatmap in Fig. 1). These were consistently labelled as chimeric composite genes (ChiC genes). Only seven ChiC genes corresponded to a gene family among the 1089 laterally acquired bacterial genes described by Nelson-Sathi [5]. This limited overlap indicates that ChiC genes are bona fide genetic innovations (Additional file 1) and point to an additional significant bacterial contribution to the evolution of Haloarchaea. Taxonomic assignment of these bacterial components by BLAST comparisons suggests that many independent bacterial sources might have been donors of these recycled fragments (Additional file 2: Figure S1). The BLAST hits for 167 composite gene components were only from one phylum, archaea, or bacteria, making their taxonomic assignment clear.

**Table 1:**
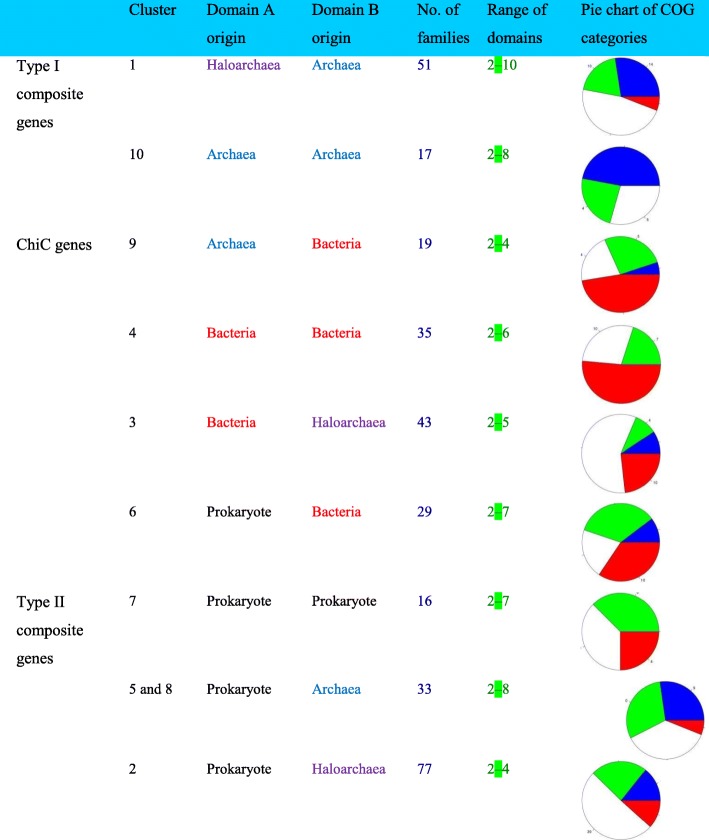
Classification of the 320 composite families found in Haloarchaea according to their component (domain) origins. *Pie charts* correspond to the distribution of COG functional annotations of the composite families for each class (*blue*: information storage and processing, *red*: metabolism, *white*: poorly characterized, *green*: cellular processes and signaling)

To test the validity of our BLAST-based taxonomic assignment, an additional phylogenetic screen was implemented. Remarkably, 99% of the phylogenetic taxonomic assignments were consistent with BLAST assignments. Of BLAST and phylogenetic taxonomic assignments, 56% match exactly, while 42% of components were assigned a prokaryote origin using one method that is resolved as either archaeal or bacterial by the other method; reflecting varying levels of resolution in the methodology rather than conflicting results (Additional file 3). Finally, clusters 2, 5, 7, and 8 correspond to 136 families of class II composite genes, built upon components of prokaryotic origins (i.e. components similar to prokaryote genes, but that we cannot assign only to Archaea or only to Bacteria according to our BLAST parameters). Many components annotated as having a prokaryote origin in the BLAST screen were also assigned a prokaryotic origin in the phylogenetic screen (80 components). However, some components considered as prokaryotic were suggested to have a bacterial (94 components) or archaeal origin (72 components) by the phylogenetic screen, suggesting that the class II composite genes may contain additional bona fide ChiC genes with components of bacterial origins that could not be detected using our methodology.

### ChiC genes are significantly involved in metabolism

**Fig. 2:**
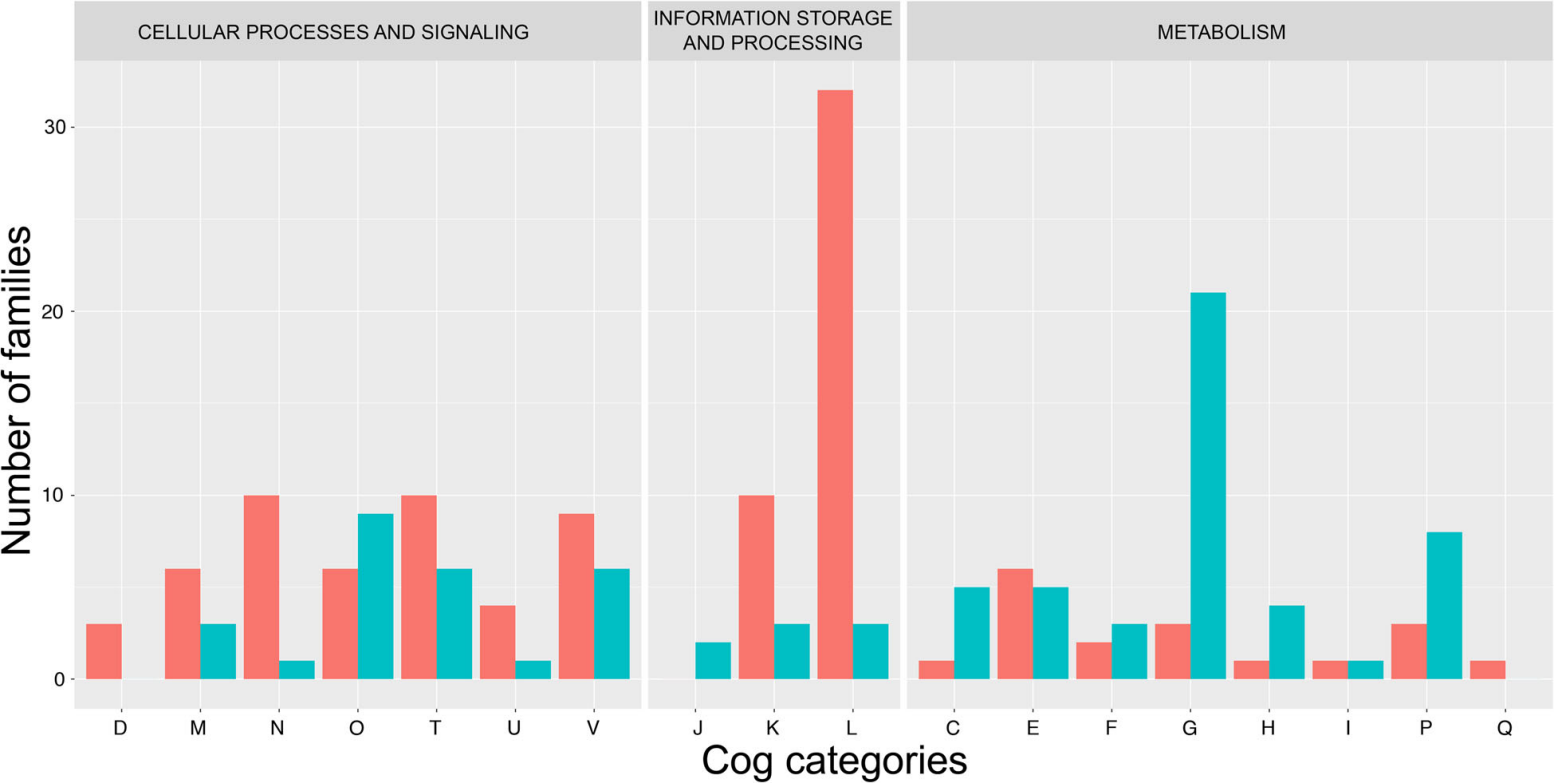
*Barplot* of functional annotation of the 126 ChiC-gene families (*blue*) and other composite families (*red*). D: Cell cycle control, cell division, chromosome partitioning, A: RNA processing and modification, C: Energy production and conversion, M: Cell wall/membrane/envelope biogenesis, B: Chromatin structure and dynamics, E: Amino acid transport and metabolism, N: Cell motility, J: Translation, ribosomal structure and biogenesis, F: Nucleotide transport and metabolism, O: Post-translational modification, protein turnover, and chaperones, K: Transcription, G: Carbohydrate transport and metabolism, T: Signal transduction mechanisms, L: Replication, recombination and repair, H: Coenzyme transport and metabolism, U: Intracellular trafficking, secretion, and vesicular transport, I: Lipid transport and metabolism, V: Defence mechanisms, P: Inorganic ion transport and metabolism, W: Extracellular structures, Q: Secondary metabolites biosynthesis, transport, and catabolism, Z: Cytoskeleton

**Fig. 3:**
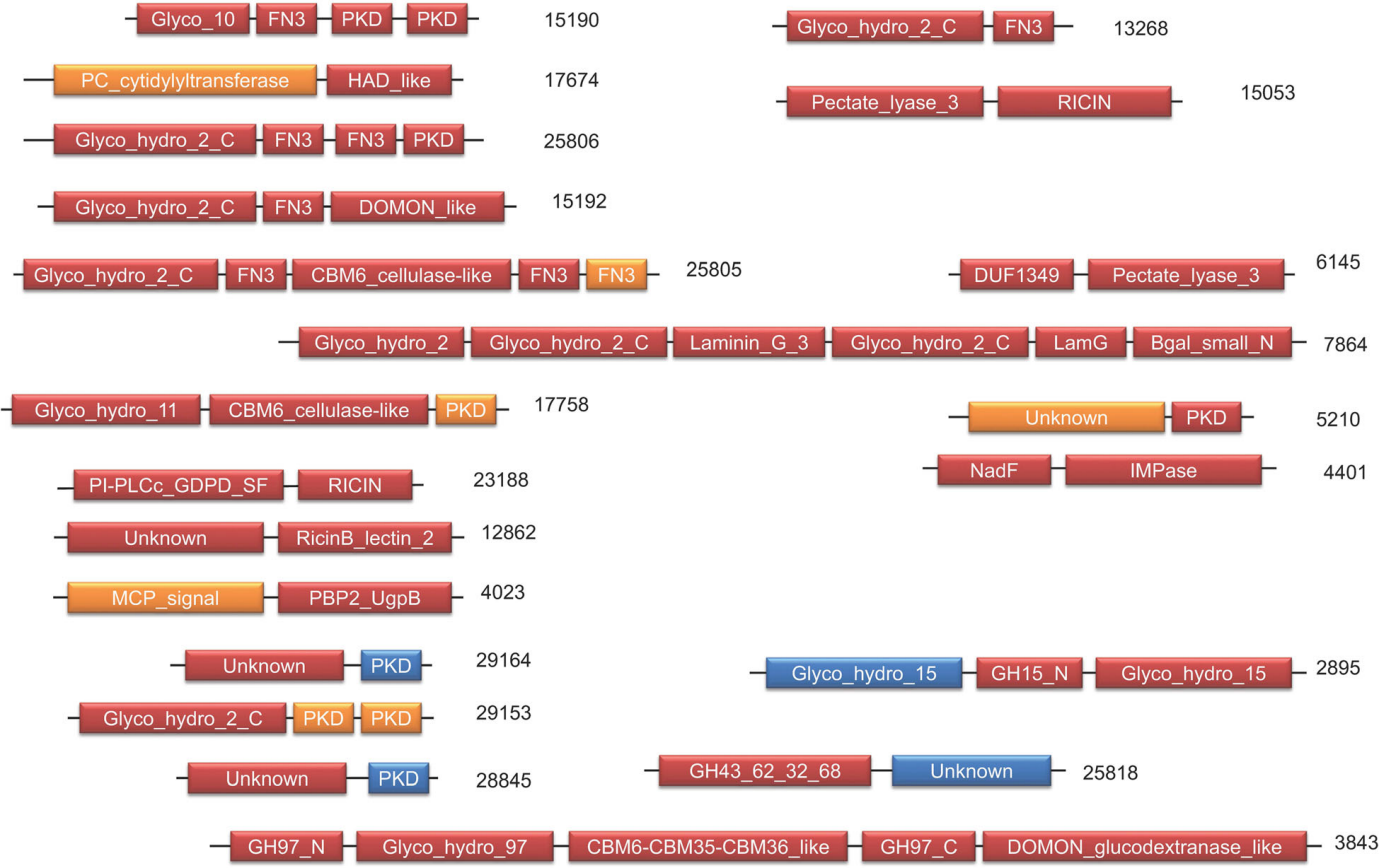
Domain architecture and origin of the 21 ChiC-protein families involved in carbohydrate transport and metabolism (*red*: Bacteria, *blue*: Archaea, *orange*: Prokaryote)

Functional analysis indicated that the 126 ChiC genes do not play the same roles as other composite genes in the cell (Fig. 1, chi-squared test, *P* = 9.263e-08). ChiC genes are enriched in metabolic functions (47 out of 126 ChiC-gene families, one-sided Fisher’s test, *P* = 1.681e-09). This result adds further evidence that bacteria contributed to metabolic functions of Haloarchaea [1, 5] and that metabolic bacterial genes can be generally recycled in genetic mergers [20]. More precisely, all metabolic categories are over-represented in ChiC genes with respect to the two other major classes of chimeric genes, except for the Q (“Secondary metabolites biosynthesis, transport and catabolism”) and E (“Amino acid transport and metabolism”) categories (Fig. 2). ChiC-gene families are particularly involved in carbohydrate transport and metabolism (G category in Fig. 2) (Fisher’s exact test, *P* = 1e-06). The large majority of the 21 ChiC-gene families in this category (18 out of 21) encode multidomain proteins carrying a glycoside hydrolase domain, such as cellulase [21], with one or several extracellular domains involved in protein-carbohydrate interaction such as fibronectin type 3 (FN3), polycystic kidney disease (PKD), ricin-like or carbohydrate binding module related domains (Fig. 3). Some of these proteins are likely secreted, as suggested by the Twin-Arginine Translocation (TAT) signal sequence detected in families 25,806 and 29,153 (Additional file 1) [22] and lack of predicted lipo-box motifs associated with membrane-anchored proteins [23, 24]. Indeed, one predicted composite gene is a part of the TAT export machinery (family 1546). These results are consistent with a change in lifestyle (from autotrophy to heterotrophy), but also with the recent finding showing that halophilic organisms can use complex carbohydrates [21]. The sparse taxonomic distribution of these 21 ChiC families suggests that utilization of complex carbohydrates probably evolved multiple times during haloarchaeal evolution, either by the strategy of domain recycling (Fig. 3) or by transfer of ChiC genes between Haloarchaea.

### Conserved composite gene families contain genes involved in salt and aerobic lifestyle

**Fig. 4:**
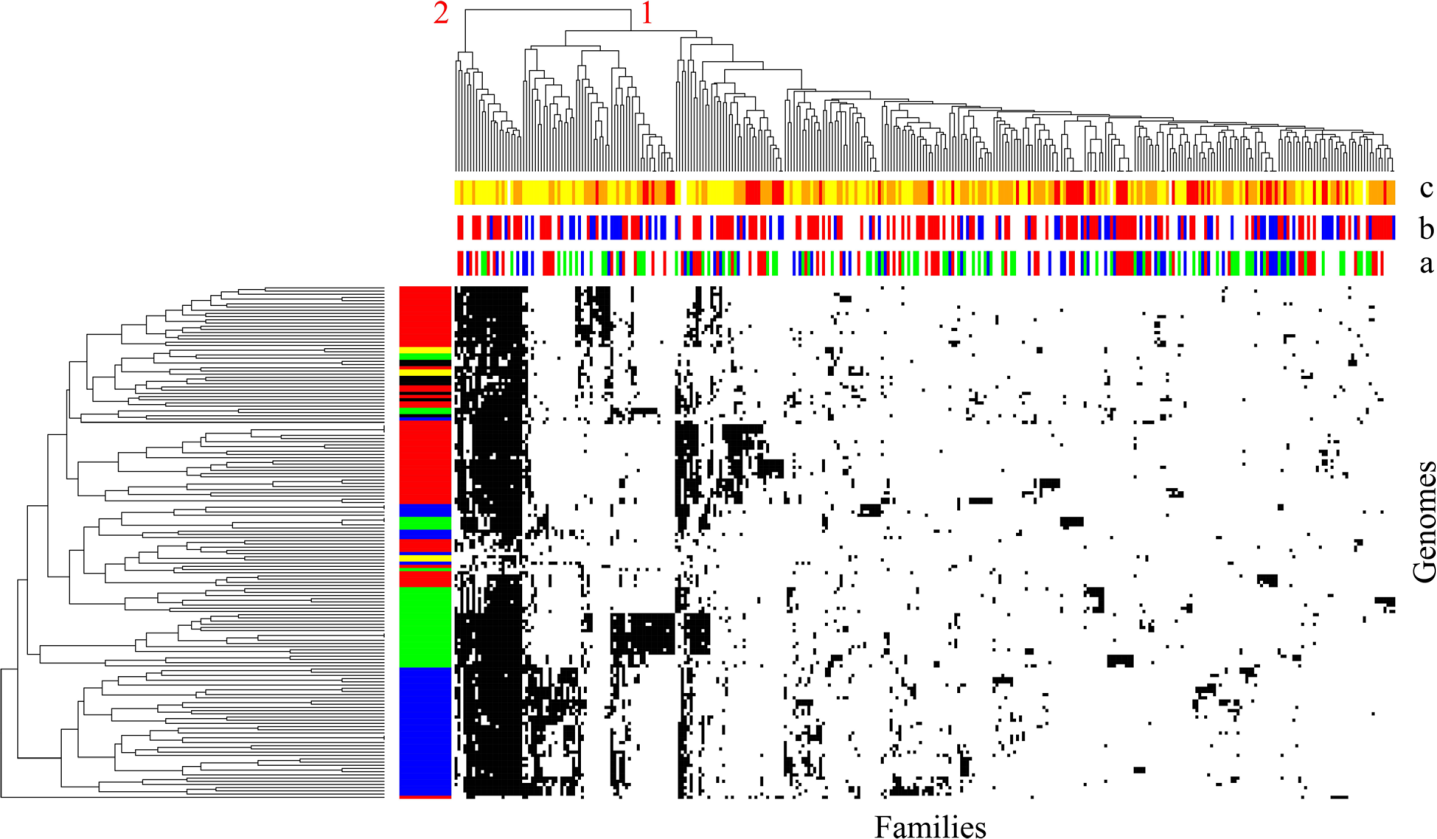
Distribution of the 320 composite gene families in Haloarchaea. The *heatmap* represents the presence (*black line*) or absence (*white line*) of a given composite gene family in Haloarchaea genomes (each line represents a given genome, each column represents a gene family). Haloarchaea genomes are colored with respect to their classification into major clades according to the study by [26] (*red*: clade B, *blue*: clade A, *green*: clade C, *yellow*: clade D, and *black*: unassigned). The *colored horizontal top bar* (**a**) indicates the mean percentage of protein identity of each gene family (*red* > 80%, *orange* > 60%, *yellow* > 40%, *white* > 25%). The *colored horizontal top bar* (**b**) indicates the type of composite family (*red*: clusters 3, 4, 6, and 9, *blue*: clusters 1 and 10, *white*: clusters 2, 5, 7, and 8). The *colored horizontal top bar* (**c**) indicates the functional annotation of the gene families according to COG categories (*red*: metabolism, *blue*: information storage and processing, *green*: cellular processes and signaling, *white*: poorly characterized). A hierarchical clustering has been performed both on columns and rows using the Jaccard distance and a complete linkage method. The hierarchical clustering of the protein families (*columns*) highlights two distinct sets of proteins, proteins that are widespread (2) and those with a sparse distribution (1)

The distribution of the 320 composite families across haloarchaeal genomes shows that most novel composite gene families (293 gene families) are sparsely distributed (Fig. 4). Interestingly, this sparse taxonomic distribution is not random with respect to currently recognized groups of Haloarchaea. We used the Mantel test (*P* = 0.001) [25] to confirm that composite genes were mainly shared by multiple genomes from the same haloarchaeal groups defined by [26]. However, it is important to note that the distribution of these composite genes is not strictly group-specific: while composite genes are mostly shared by related genomes, only 120 of the 293 gene families are fully specific to a single haloarchaeal group. This narrow taxonomic distribution suggests that the gene families may be more recent inventions; however, we cannot discount the possibility that they are ancient acquisitions that have been lost in multiple lineages. The distribution of the remaining 173 gene families in multiple major haloarchaeal groups suggests that they were either acquired in a common ancestor of these groups and subsequently differentially lost, or that they have been laterally transferred within the haloarchaea. This general consistency of the distribution of composite genes with the proposed haloarchaeal phylogeny suggests that composite genes have persisted in these groups for a certain period of time, and therefore likely provide adaptive value to them. Otherwise, these novel genes are unlikely to have been fixed in these genomes [27].

Remarkably, a minority of composite gene families exclusive to Haloarchaea are broadly distributed across the Haloarchaea (two set of proteins in Fig. 4, 23 families). Genes within these families also show a larger divergence in primary sequences (measured in percentage identity between pairs of homologous sequences). Protein identify of 50.16% is the median for the broadly distributed genes vs 63.09% for the other composite genes (two-sample Wilcoxon test, *P* = 0.0008646). Taken together, their broad taxonomic distribution and the accumulation of substitutions in their sequences suggest that these composite genes, exclusive to Haloarchaea, are ancient and were possibly invented during haloarchaeal genesis.

Recent debate has centered around the finding that major archaeal groups, including the Haloarchaea, were underpinned by large scale lateral gene transfers at their origins [5, 6]. A reanalysis of this dataset has argued that the acquisition of genes by LGT may have been a more piecemeal process [7]. However, the methodological basis of this reanalysis has very recently been challenged as artificially inflating the number of more recent events [28]. We tested whether the composite genes reported in our study could provide additional (although clearly distinct) elements to the debate regarding the tempo of acquisition of bacterial gene fragments into Haloarchaea. Our dataset of composite gene families has no overlap with the LGTs identified in the study focusing on full-sized genes if bacterial origins [6, 7] and has very limited overlap with LGTs identified in other recent studies [5, 9] (Additional file 1). Instead, our dataset represents a novel contribution of gene families to Haloarchaea created by gene rearrangement, some of which include fragments of bacterial origin. Although we find no significant evidence for a single acquisition of our ChiC-gene families at the origin of Haloarchaea (*P* = 0.202 using the test for monophyly supplied by authors of [6]), for this new set of haloarchaeal gene families, we find evidence for a combination of both ancient and potentially more recent inventions. The invention of “new genes” can be a driver of phenotype evolution over even relatively short evolutionary distances [14]. While this is true for both ancient and more recent acquisitions, gene families that are more broadly conserved in prokaryotes (or within haloarchaea) are more likely to be essential to that group, given the rapid loss of unessential genes in the compact genomes of prokaryotes [29]. Thus, focusing on their functions, as well as on their distribution, may help us to understand how the first haloarchaea tackled the challenges of adaptation to an aerobic and salty environment.

Haloarchaea have switched from an anaerobic autotrophic methanogenic ancestral state to their current derived method of energy production, heterotrophic aerobic respiration [5, 21]. Two broadly distributed composite genes that were plausibly invented during the haloarchaeal genesis are involved in redox activities, required for the electron transport in aerobic respiration: families 1776 and 1784. The former is a ChiC-gene family and encodes proteins carrying two conserved domains: the N-terminal domain of bacterial origin is characterized as either a putative heme peroxidase domain (2.20e-113) or a Chlorite dismutase (1.11e-83), and a C-terminal domain of prokaryotic origin is characterized as either an antibiotic biosynthesis monooxygenase (ABM - 1.06e-09) or a Heme-degrading monooxygenase (HmoA – 6.67e-09) [30]. In spite of its broad distribution and its experimentally defined essential nature in the model organism *Haloferax volcanii* (accession: HVO_1871) [31], the molecular role of family 1776 is not known. Heme and heme-like molecules are easily oxidized and reduced, suggesting this gene may play a role in the electron transport chain for aerobic respiration. Another family with a putative similar role is family 1784 annotated as cytochrome b subunit of the bc complex involved in energy production and conversion. This gene is a major component of electron transport for generating a proton motive force. In mitochondria, it is utilized during aerobic respiration, though in prokaryotes it can also be used in anaerobic respiration (e.g. denitrification). Though many methanogens and all *Methanosarcinales* utilize cytochromes for conservation of energy when growing on CO_2_ and H_2_ [32], the identified haloarchaeal cytochromes are not directly related to them, implying a different or nuanced functional capacity. In line with this interpretation, additional electron transport genes of bacterial origin have been observed in haloarchaeal genomes [4]. The idea that these broadly distributed novel composite genes may have been an important feature in the transition of Haloarchaea from anaerobic to aerobic environments is supported by the large number of additional sparsely distributed composite gene families with putative redox roles, including Pyrrolo-quinoline quinone and redoxin (families 17,613, 12,148, 13,590, 14,246, and 18,015).

One of the major challenges of living in a salty environment is how to regulate osmotic pressure. Haloarchaea utilize a salt-in strategy: they export Na^+^ ions and pump in K^+^ to molar concentrations to counter the osmotic pressure necessary for living in saturated brines [33]. This strategy, though not unique to the Haloarchaea [34], is found throughout the Haloarchaea and was likely in their common ancestor. Two conserved composite families may play a role in osmotic stress/balance. Family 1329 encodes ChiC proteins carrying a TrkA domain of bacterial origin on the C-terminus and a domain of unknown function in N-terminus. Analysis in *Haloferax volcanii* (HVO_2617, 403aa) indicates the gene products are three-pass integral membrane proteins in the N-terminus. Homology with the TrkA domain in *Escherichia coli* suggests the protein is involved in potassium ion uptake. The other family involved in salt-in strategy and widespread throughout the Haloarchaea is family 1906. This modular Class II composite gene encodes a two-domain protein annotated as an inorganic ion transporter, as it contains Na^+^/H^+^ antiporter MnhE subunit domains of prokaryotic origin coupled with a divergent universal stress protein (USP) domain with no significant sequence similarity outside haloarchaea. In total, 11 putative composite genes are assigned to the inorganic ion transport and metabolism COG category, of which three are broadly conserved across the group and eight are putative ChiC genes (Fig. 2). None of the composite gene families identified in this study were significantly regulated in response to salt concentration in previous transcriptome studies [35–37]. However, the predicted function of these families suggests that their acquisition may have been crucial to the adaptation of haloarchaea to hypersaline environments and the salt-in strategy.

### Class I and class II composite genes and ChiC genes code for proteins optimized for life in salty environments

**Fig. 5:**
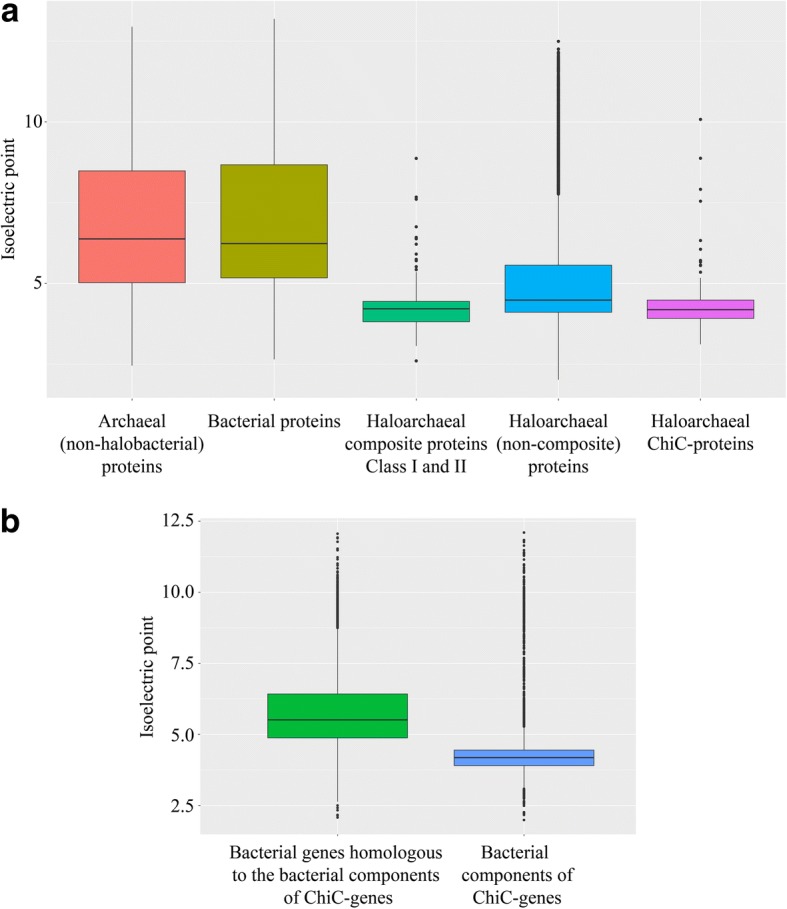
**a** *Boxplots* showing the distribution of isoelectric points of proteins according to their origins and their types. The boxplot indicates the median line, first and third quartiles. Outliers that are 1.5× above the upper quartile or below the lower quartile are indicated as *dots*. **b** *Boxplots* showing the distribution of the isoelectric points of components originated from bacteria. Bacterial components correspond to bacterial genes which aligned with the ChiC-gene components assigned as of bacterial origin. The boxplot indicates the median line, first and third quartiles. Outliers that are 1.5× above the upper quartile or below the lower quartile are indicated as *dots*

The salt-in strategy means that haloarchaeal proteins require additional adaptation to remain soluble in hypersaline conditions and almost all haloarchaeal proteins have a decreased isoelectric point [33]. In order to assess the long-term presence of class I and class II composite genes and ChiC genes in haloarchaeal genomes, we calculated their isoelectric points. Isolectric points of class I and class II composite genes and of ChiC genes do not differ from that of the rest of the haloarchaeal proteins and are significantly lower than that of other archaeal and bacterial proteins (Fig. 5; Wilcoxon test, *P* < 2.2e^−16^). For ChiC genes, these lower isoelectric points are likely the result of a process of genetic optimization of their acquired bacterial genetic fragments in Haloarchaea, since their bacterial homologues have higher isoelectric points. Consistently, there is a significant difference (Wilcoxon test *P* < 2.2e^−16^) in isoelectric points between the top five bacterial sequences matching with the bacterial components of these ChiC genes and the bacterial components of the ChiC genes (Fig. 5). Thus, amino acid compositional changes confirm the significant time of residency, and likely adaptive role, of these novel genes in these halophiles.

## Conclusions

Our network analyses identify 320 novel composite genes that evolved in Haloarchaea. At least 24 such gene families likely appeared early in the evolution of Haloarchaea, possibly during their genesis, and were largely conserved since that time, suggesting that they may play essential roles in the group. A total of 296 additional composite gene families either appeared later, in already diverged haloarchaeal groups, or were acquired in the common ancestor of haloarchaea and subsequently lost in different haloarchaeal lineages. Importantly, 126 of all 320 novel composite gene families were derived from genetic material from bacterial genomes. These haloarchaeal ChiC genes unravel a substantial additional bacterial contribution to the evolution of Haloarchaea, in addition to the many reported cases of LGT from bacterial donors. These novel composite genes were more than transient inventions in a few haloarchaeal genomes: these novel composite genes were optimized to code for proteins with low isoelectric points and are distributed in multiple related genomes suggesting that composite and ChiC genes certainly play a role in the biology of Haloarchaea. Haloarchaeal ChiC genes are largely involved in metabolic functions and many of these functions are relevant in the adaptation of Haloarchaea to an aerobic lifestyle. Further work would be required to assess whether these metabolic functions are essential for Haloarchaea and we expect that this is more likely to be true for composite genes that are broadly conserved in the group. Contrasting this, composite genes with archaeal components are enriched in informational functions in DNA replication and repair. The contribution of ChiC genes to operational functions and archaeal composite genes to informational functions draws interesting parallels to patterns observed in eukaryogenesis, where bacterial genes also largely contributed to operational functions and archaeal genes contributed to informational functions in a chimeric lineage [38].

Overall, our work encourages a systematic search for novel composite and ChiC genes across all archaeal major groups, in order to better understand the origins of novel group-specific prokaryotic genes, and in order to test to which extent archaea might have adjusted their lifestyles by incorporating and recycling laterally acquired bacterial genetic fragments into new archaeal genes.

## Methods

### Dataset creation

We assembled a protein sequence database by downloading every archaeal genome from the NCBI Genome database in April 2016 (803 genomes, 1,816,486 proteins) (Additional file 4). 2078 eubacteria genomes annotated as complete according to the NCBI Genome database (7,239,663 proteins) (Additional file 4).

### Construction of gene families

Proteins were clustered into families using the same method as [18, 38]. The 1,816,486 archaeal protein sequences were compared pairwise using BLASTP [39] (version 2.2.26) (E-value threshold < 1e-5 and using the soft-masking parameter for low complexity regions). Pairs of proteins that can be aligned > 80% of their length and show protein identities > 30% were kept to construct an undirected graph. In this graph, each node corresponds to a sequence and two nodes are linked if the corresponding sequences show a BLAST hit with an E-value < 1e-5, a sequence identity > 30%, and a mutual sequence coverage > 80%. Connected components in this graph were considered protein families. The archaeal protein sequences were compared to 7,239,663 eubacterial protein sequences using BLAST. Families that only included halobacterial proteins from at least three distinct genomes and that had no global similarities (hence no homology) with any eubacterial sequences (mutual coverage > 80%, sequence identity > 25%) were retained for ChiC-gene detection.

### Domain and functional annotations

Domains were predicted using the conserved domain database (CDD) (version 3.13) [40] (default parameters). Sequences were functionally annotated with the halobacteria profiles dataset from the EggNog database (version 4.5) [41] (default parameters). For each family, if > 60% of gene members share the same EggNog annotation, this EggNog annotation has been assigned to the family, if not, the family function was considered as unknown. Cellular localization was investigated using the PSORTdb (version 3.0) [42] (default parameters for archaea). For each family, the more abundant localization annotation has been used as family localization.

### Detection and origin assignment of component families

For each retained sequence, component sequences were clustered into component families according to the following rule: if two component sequences overlapped by > 70% of their lengths on the protein composite, they belonged to the same component family. A refining procedure has been done in order to merge overlapping and/or nested components families: two component families were merged if one component family is included by > 70% of its length into the other one.

Component families were assigned an origin based on their taxonomic composition. If the five best prokaryotic component sequences, according to their BLASTP bitscore against the composite gene, matched with the same prokaryotic domain/phylum (e.g. Archaea or Bacteria for domain assignment), we considered the component to have originated from that prokaryotic domain/phylum. If the component family contained < 5 sequences, or if archaeal and bacterial sequences were both present among the five best sequences, we considered the component to originate from prokaryotes.

To explore whether use of the top five BLAST hits was a good proxy for assessing component origin, phylogenies were generated for all composite gene domains and their corresponding component families. Sequences were aligned using MAFFT [43]. A HMM profile was constructed for each alignment and used as a query to search the gene family including directly annotated composite genes [44]. This search was used to identify components within the composite gene family that were not directly detected using BLAST and add their sequence to the dataset. The final dataset was aligned using MAFFT, regions of uncertain alignment were trimmed using trimAL in automated 1 mode [45], and phylogenies were inferred using the LG + G model implemented in IQ-tree [46, 47]. Trees were manually screened to infer the origin of composite components using the following criteria: if archaeal components from outside the Haloarchaea form a strongly supported clan (bootstrap support > 70%) with the composite gene component nested within that clade, the domain is considered to be of archaeal origin. If the composite gene domain is nested in a strongly supported clan with bacterial components the domain is considered to have a bacterial origin. If any of these criteria are not met, then a domain is considered prokaryotic. All sequences, alignments, and phylogenies are available at https://figshare.com/s/906f41485528e4a99173.

The test used for comparison of sets of phylogenies described in [6] was kindly supplied by its authors. Sequences from each haloarchaea specific single copy gene family that included at least four haloarchaeal taxa were aligned using MUSCLE with default settings [48], trimmed using trimAL in automated1 mode [45], and phylogenies were inferred using the LG + G model implemented in IQ-tree [46, 47]. Phylogenies inferred from ChiC-gene families were used as the “import” gene set and haloarchaea-specific gene families that were not identified as LGTs in previous studies [5, 6, 9] were used as the reference gene family set.

### Detection of composite genes and ChiC genes

Genes were defined as composite genes if they had at least two components detected, or if they had one component plus at least one domain annotation, on a region that was non-overlapping with the detected component. When the component was of bacterial origin, the composite gene was considered as a ChiC gene. Sequences for all composite gene families are available at https://figshare.com/s/778c566b568c24d9ec83.

### Isoelectric points calculation

Isoelectric points were calculated using the Isoelectric Point Calculator [49].

## Additional files

Additional file 1: Annotation of the 320 ChiC-gene families detected. (XLSX 37 kb)

Additional file 2: **Figure S1.** *Pie chart* of bacterial affinities of the bacterial components of ChiC-gene families. For each bacterial component of ChiC genes, we looked at the phylum to which its five top hit sequences belong. The origin was assigned to a specific phylum only if the top five hit sequences belonged to the same bacterial phylum. The majority of ChiC genes contain bacterial components with no clear origin at the phylum level as they do not meet this criterion. Of the 35 ChiC genes with a BAC-BAC structure, only three include multiple components with the same predicted phylum origin. (PNG 86 kb)

Additional file 3: BLAST and phylogenetic taxonomic assignments of composite gene family components. (XLSX 23 kb)

Additional file 4: List of the 803 archaeal genomes and the 2078 bacterial genomes we used in our comparative analysis. (XLSX 206 kb)
