# Supplementary figures and images for: Hundreds of novel composite genes and chimeric genes with bacterial origins contributed to haloarchaeal evolution
Source: Genome Biol. 2018 Jun 7;19:75. doi: 10.1186/s13059-018-1454-9 (PMC5992828; doi:10.1186/s13059-018-1454-9)

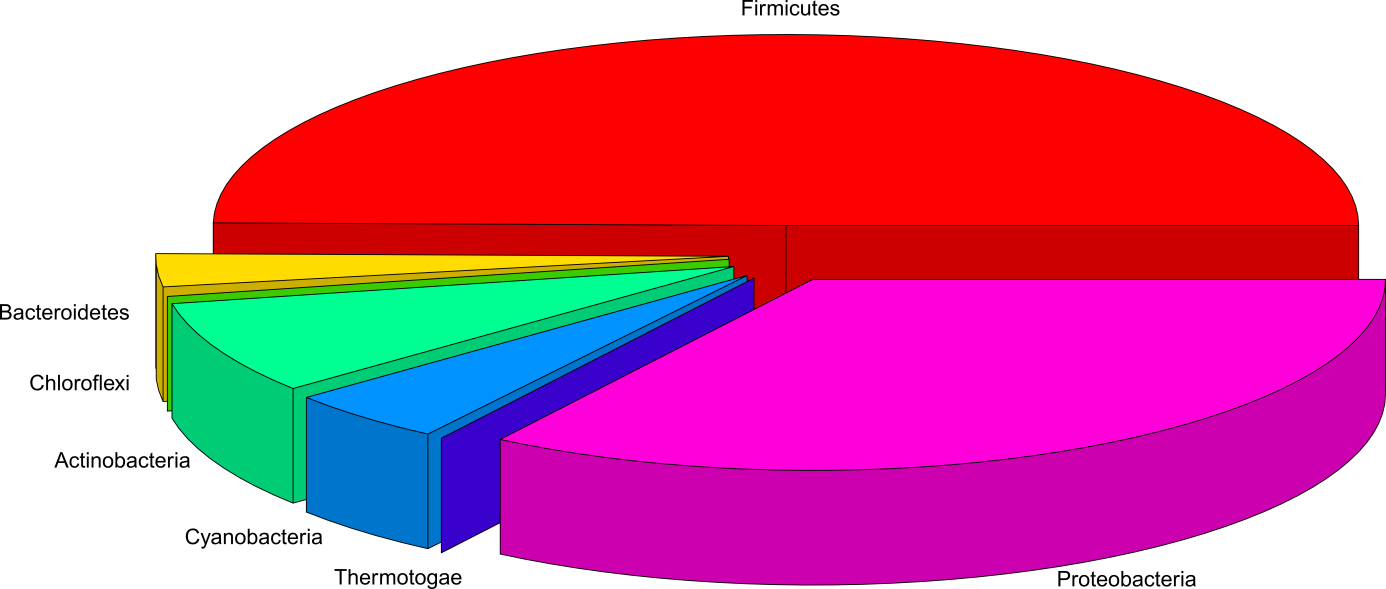

Supplement: Supplementary file 2 — Figure S1. Pie chart of bacterial affinities of the bacterial components of ChiC-gene families. For each bacterial component of ChiC genes, we looked at the phylum to which its five top hit sequences belong. The origin was assigned to a specific phylum only if the top five hit sequences belonged to the same bacterial phylum. The majority of ChiC genes contain bacterial components with no clear origin at the phylum level as they do not meet this criterion. Of the 35 ChiC genes with a BAC-BAC structure, only three include multiple components with the same predicted phylum origin. (PNG 86 kb) [file 13059_2018_1454_MOESM2_ESM.png]
